# Impact of Atopic Dermatitis on the Quality of Life of Children in Ethiopia: A Multicenter Prospective Cohort Study

**DOI:** 10.3390/children13020201

**Published:** 2026-01-31

**Authors:** Abraham Getachew Kelbore, Wendemagegn Enbiale, Jacqueline M. van Wyk, Efa Ambaw Bogino, Aldo Morrone, Anisa Mosam

**Affiliations:** 1 Department of Dermatology, College of Health Sciences and Medicine, Wolaita Sodo University, Wolaita Sodo P.O. Box 138, Ethiopia; 2 Department of Dermatology, Nelson R. Mandela School of Medicine, University of KwaZulu-Natal, Durban 4001, South Africa; 3 Department of Dermatology, College of Health Sciences and Medicine, Bahir Dar University, Bahir Dar P.O. Box 79, Ethiopia; 4 Collaborative Research and Training Center for Neglected Tropical Diseases, College of Medicine and Health Sciences, Arba Minch University, Arba Minch P.O. Box 21, Ethiopia; 5 Department of Clinical and Professional Practice, Nelson R. Mandela School of Medicine, University of KwaZulu-Natal, Durban 4041, South Africa; 6 Department of Health Sciences Education, Faculty of Health Sciences, University of Cape Town, Cape Town 7925, South Africa; 7 International Institute Social, Medical, and Anthropological Sciences (IISMAS), 00118 Rome, Italy; 8 Saint Camillus International University of Health and Medical Sciences, 00131 Rome, Italy; 9 Inkosi Albert Luthuli Central Hospital, Durban 4091, South Africa

**Keywords:** atopic eczema, pediatrics, SCORAD index, quality of life, hospital-based, Ethiopia

## Abstract

**Highlights:**

**What are the main findings?**

- Standard treatments combined with educational interventions effectively reduce disease severity and quality of life impairments in children with AD after six months of follow-up.
- The severity of AD (according to SCORAD score), changes in SCORAD scores, age at disease onset, and the number of children in the family were independent predictors of quality of life.

**What are the implications of the main findings?**

- Identified predictors of quality of life can guide more personalized management for children with AD.
- Integrated clinical and psychosocial care approaches for pediatric AD are crucial in resource-limited settings.

**Abstract:**

Background: Atopic dermatitis (AD) is a chronic, pruritic and relapsing inflammatory skin disorder affecting children’s quality of life (QoL). Despite rising global prevalence, data on its impact on QoL in low-resource settings remain limited. This study aimed to assess the impact of AD and associated factors on the QoL of children and assesses the effect of educational intervention in Ethiopia. Methods: A prospective cohort study was conducted among 461 AD children and their caregivers across four randomly selected hospitals dermatology clinics in Ethiopia from October 2022 to March 2024. Assessments included AD Severity using Scoring Atopic Dermatitis (SCORAD), Infants’ Dermatitis Quality of Life Index (IDLQI) for children aged 0–4, and Children’s Dermatology Life Quality Index (CDLQI) for children aged 5 to 16. Participants received educational guidance from trained nurses during follow-up beyond routine AD treatment. Trained personnel collected clinical and sociodemographic data. AD severity and QoL were reassessed after 6 months. Descriptive, univariate, and linear regression analyses identified factors influencing QoL, with associations reported as odds ratios (95% CI) and significance set at *p* < 0.05. Results: Of 461 children, 424 (92%) completed follow-up. Most were under five (67%) with a median age of 3 years; 72.2% had AD onset before age two. Most caregivers were female (68.9%). After six months, clinical signs of AD, including dryness, erythema, excoriation, and lichenification, improved notably. Mild AD increased by 33.5%, while moderate and severe cases decreased by 17.5% and 16%, respectively. QoL significantly improved across all domains (*p* = 0.001). Baseline disease severity (β = 0.11), change in severity (ΔSCORAD) (β = 0.043), number of dependents (β = −0.71), and age at disease onset (β = 0.005) as significant predictors of QoL. Conclusions: AD significantly impairs QoL in Ethiopian children, with greater severity causing more disruption. Routine treatments with educational interventions significantly improve disease severity and QoL. Integrated clinical and psychosocial care approaches for pediatric AD are crucial in resource-limited settings.

## 1. Introduction

Atopic dermatitis (AD) is the most common chronic inflammatory skin disorder in children and adolescents [1]. It is characterized by relapsing episodes of intense pruritic and eczematous lesions. And it is often associated with other atopic conditions such as asthma or allergic rhinitis, and due to the nature of the disease’s interference with daily functioning, the quality of life of the children with the impairment ranges from mild to extremely severe [2,3]. Globally, the prevalence of AD in children varies widely, ranging from 15% to 20% in many countries [4,5,6]. In Ethiopia, the regional prevalence rates vary from place to place: 4.4% at Jimma [7], 9.6% at Mekelle [8], 11.3% at Wolaita Sodo [9], and 21.3% in Addis Ababa [10], with lower prevalence rates reported among children in rural areas and children from lower socioeconomic classes.

Recent studies have reported an increase in the prevalence of AD worldwide, which has been attributed to changes in lifestyle, nutrition, and other environmental factors like urbanization, pollution, and climate change [11]. Furthermore, variation in prevalence among populations of similar ethnic backgrounds living in different geographic or socioeconomic settings suggests that environmental triggers play a significant role in disease development [12].

AD is a multifactorial disease involving a complex interplay between immune dysregulation, epidermal gene mutations, and environmental factors [13], clinically characterized by itchy, recurrent, and dry skin and a rash that may become lichenified with flexural involvement as the disease progresses. The nature of the diseases is chronic and interferes with routine daily functioning; the QoLof children and adolescents can be affected [14]. Beyond cutaneous manifestations, persistent itching, especially in infants, can profoundly disrupt sleep, emotional well-being, and overall quality of life for both the child and their caregivers [15].

Sleep studies show that children with AD often wake up from scratching and sleep less efficiently than healthy children. This cycle of discomfort and poor sleep impacts their overall well-being and daily life [16,17]. It is well known that the growth and development of infants and young children mainly occur during sleep, and the itching of AD will directly lead to a lack of sleep in children, affect sleep quality, and thus indirectly affect growth and development. Among older children, the visibility of AD lesions and related social stigma may adversely affect self-esteem and peer relationships and have been associated with suicidal ideation, with chronic scratching reported as a possible contributing factor [18,19].

The degree to which AD affects a child’s QoL is closely related to the severity of the disease [20]. Studies have shown that children with severe AD are more likely to experience treatment non-adherence, corticosteroid phobia, and resort to unproven alternative remedies, often due to limited understanding of the disease or lack of proper counselling [21,22]. Although numerous studies have examined the impact of AD on quality of life in other countries [23,24,25,26], no such research has been conducted in the Ethiopian context. This constitutes a significant knowledge gap, especially given region-specific socioeconomic, healthcare access, and environmental influences that may modulate the disease expression and its psychosocial consequences.

Moreover, AD has been shown to have a greater impact on QoL than many other chronic conditions. Studies comparing children with dermatological and general medical illnesses have found that AD is the leading skin disease associated with QoL decline. In fact, the reduction in QoL among children with AD is comparable to that experienced by children with renal diseases and more severe than that reported for conditions such as asthma, psoriasis, cerebral palsy, cystic fibrosis, enuresis, and epilepsy [27,28]. The chronic itch of AD is not only a physical distress but has been strongly linked to mental health challenges, including depression, anxiety, sleep disorders, and irritability [29].

Given the high burden of AD on children’s physical, emotional, and social well-being, and the evidence of its impact on QoL, it is crucial to understand these effects in the Ethiopian context. This study, therefore, aims to evaluate the effect of disease severity and educational intervention on the QoL of children with AD across multiple regions of Ethiopia. The findings will provide crucial insight to guide patient-centred care, inform clinical practice, and shape national strategies aimed at improving dermatological outcomes and overall well-being in pediatric populations.

## 2. Materials and Methods

### 2.1. Study Design and Settings

We conducted a multicentre prospective cohort study between October 2022 to March 2024 in four public hospitals: two comprehensive specialized hospitals (Wolaita Sodo University and Nigist Eleni Mohammed Memorial) and two general hospitals (Arbaminch and Dr. Bogalech Gebre Memorial) across Central and Southern Ethiopia regional states, Ethiopia, including areas formerly part of the Southern Nations, Nationalities, and Peoples’ Region (SNNPR). Central Ethiopia has three special districts and seven zones, while South Ethiopia comprises twelve zones [30,31]. According to the 2021/2022 Regional Health Bureau report, the area includes 76 government hospitals, 723 health centres, and 3874 health posts [32].

Nationwide, there are fewer than 200 dermatology professionals. Public healthcare operates under a three-tier system: primary (health posts and centres), secondary, and tertiary. Only nine hospitals offer dermatology services in the two regions studied, staffed by just 14 dermatologists, making access and availability very limited [33].

### 2.2. Study Population

All children 16 years of age and younger diagnosed with AD, together with one selected family member or caregiver associated with them during the study period. In this study, a caregiver is an individual, irrespective of biological or familial relationship, who provides care and support to an ill child within the home or anywhere else.

The study recruited all children diagnosed with AD based on the UK Working Party Diagnostic Criteria for Atopic Dermatitis, which has been validated for use with Ethiopian children [34]. Depending on the clinical presentation and morphology of the skin lesions, children diagnosed with AD received treatment accordingly: topical corticosteroids, systemic steroids for extensive and non-responsive lesions to topical treatment in older children, emollients, and broad-spectrum antibiotics for patients with bacterial superinfections. However, in this study, biological treatments, including dupilumab, were not used. Health information about disease progression and skin care of patients with AD was given as part of an educational intervention. Children with a known psychiatric disorder, a history of steroid and antihistamine treatment intake within the last two weeks, and families or caregivers age less than 18 years were excluded.

### 2.3. Sample Size Determination and Sampling Technique

In an earlier phase of the study, the sample size of 470 was calculated based on an 11.3% AD prevalence, a 95% confidence interval, a 0.03% margin of error, and an additional 10% for potential non-responses [9,35]. Ultimately, 461 children diagnosed with AD were enrolled in the cohort follow-up for this study. Four zones were randomly selected from two regions, and hospitals within these zones providing dermatological services were purposively chosen. Based on average patient flow over the previous six months, the sample size was proportionally allocated to each hospital. Hospitals were stratified by level (secondary or tertiary), and participants were selected through a systematic sampling method.

All participants were given follow-up appointments, and parents or caregivers were contacted by phone to encourage attendance. However, 37 children could not be reached despite repeated efforts and were excluded from the final analysis. Reported reasons included long distance from the hospital (12), recurrence of illness (11), scheduling conflicts with social events (5), and unspecified reasons (9). At the six-month follow-up, the 424 remaining children were reassessed using the SCORAD index and age-appropriate quality of life tools. A total of 284 children were assessed with the IDLQI and 140 with the CDLQI, completing the study’s follow-up phase.

### 2.4. Data Collection Tools and Procedures

The sociodemographic data were collected by structured interview questions that included age, sex, residence, family (occupation, educational status, and monthly income), caregiver/family marital status, history of atopy, age of onset, and duration of the disease since diagnosis, comorbidity, and sex of caregiver/family.

QoL of children was assessed with the Infants’ Dermatitis Quality of Life Index (IDLQI) for children aged less than 5 years and the Children’s Dermatology Life Quality Index (CDLQI) for children from 5 to 16 years of age. Both questionnaires consist of ten items, all related to the week preceding the testing. The items in IDLQI explore itching, the child’s mood, sleeps issues, playtime, and the effect on family activities, mealtime, treatment, dressing, and bath time. The CDLQI consists of 10 items covering symptoms, leisure, school, personal relationships, sleep, and treatment effects, with a score range of 0–30. Higher scores indicate greater impairment. For younger children or those with reading difficulties, the questionnaire was completed with assistance from a parent or caregiver, following standard guidance for IDLQI administration.

The impact of AD on quality of life is categorized as follows: no effect (scores 0–1), small effect (2–6), moderate effect (7–12), very large effect (13–18), and extremely large effect (19–30).

We categorized it from five to three to assess the quality of life impairment. The impact of atopic dermatitis on children’s QoL before and after follow-up was assessed using the following total IDLQI/CDLQI score cut-offs: 0–6 for mild impairment, 7–12 for moderate impairment, and ≥13 for severe impairment, by subtracting the six-month IDLQI scores from the baseline scores to determine the change (∆IDLQI). Similarly, ∆CDQLI was calculated, and we used it as a dependent variable for linear regression as per the age of children.

Clinical dermatologists assessed the severity using the baseline and after six months of follow-up SCORAD index, which considers both objective (clinical presentation and extent) and subjective AD scores. Higher SCORAD values indicated higher AD severity. Follow-up visits with the dermatologists were carried out every three months. The patients were reassessed after six months. A SCORAD index result of less than 25 indicated mild disease, 25 to 50 as moderate disease severity, and greater than 50 as severe AD. A score of 0 indicates no symptoms.

Operational definition of a caregiver: someone who may or may not have a blood relationship with the child but cares for the sick child at home or anywhere else.

### 2.5. Data Collection Tool

A structured interview questionnaire was developed based on the literature review and adapted to fit the study area’s regional context. All the tools were translated from English to Amharic and then back to English by different professional translators to ensure the consistency of the information.

The pre-test was performed on 5% of the sample at the Wolaita Sodo University Comprehensive Specialized Hospital before the start of the study, and these pre-tested samples were not included in the study. All children with AD were examined by trained dermatologists, and the data collectors (nurses) received training on the data collection tools, procedures, and informed consent process before the study began. The data were collected at the dermatology outpatient unit in each of the participating hospitals.

### 2.6. Data Processing and Analysis

Collected data were double-entered into EpiData software (v4.2.0.0; EpiData Association, Odense, Denmark), cleaned using frequency and cross-tabulation checks, and exported to SPSS version 27 for analysis. Descriptive statistics were used to summarize the data, including frequencies, proportions with 95% confidence intervals (CIs), means, standard deviations, and medians.

Changes in SCORAD (∆SCORAD) and IDLQI/CDLQI (∆IDLQI/CDLQI) were calculated by subtracting the six-month scores from baseline values. The five severity categories of ∆IDLQI/CDLQI were merged into three levels: mild, moderate, and severe, and visualized using a Sankey diagram to illustrate changes over time.

The Wilcoxon signed-rank test assessed differences in children’s quality-of-life scores between baseline and six-month follow-up. Multicollinearity among candidate variables was evaluated using tolerance and Variance Inflation Factor (VIF), with no issues found.

Univariate associations were examined between baseline characteristics and IDLQI/CDLQI and ∆IDLQI/CDLQI. Multiple linear regression models included variables with a univariate *p* value < 0.25 to identify associated factors. Results with a *p*-value < 0.05 were considered statistically significant.

### 2.7. Data Quality Assurance

A pre-validated standard questionnaire was used to collect data to maintain quality. Continuous supervision was performed during data collection at each hospital, and ten percent of the dataset was double-entered to check the data’s accuracy.

## 3. Results

### 3.1. Sociodemographic Characteristics of Study Participants

A total of 461 children with AD were initially enrolled in the study; 424 (92%) completed the six-month follow-up; however, 37 participants (8%) were lost to follow-up. A total of 424 study subjects and their caregivers or families (424) were included in the final analysis.

Out of the 424 children, 231 (54.5%) were male. The age distribution ranged from 6 months to 16 years, with a median age of 3 years (interquartile range [IQR] of 1 to 5 years). Most of the children, 284 (67%), were under the age of five. The caregivers’ ages ranged from 18 to 54 years, with an average age of 30.4 years (±5.84). Most caregivers were women, 292 (68.9%), and were married, 373 (88%). A majority of the children, 253 (59.7%), lived in urban areas. Regarding parental education, 169 mothers (39.9%) and 200 fathers (47.2%) had completed tertiary education (see Table 1).

### 3.2. Clinical Characteristics Profile Among Children Diagnosed with Atopic Dermatitis

Clinical signs and symptoms of atopic dermatitis first appeared before age two in 306 children (72.2%). The most common clinical features observed at baseline included dry skin (90.3%), redness (85.6%), scratch marks (80.2%), lichenification (72.9%), swelling (63%), and oozing or crusting (54.5%).

After six months of standard treatment and educational intervention, there was a noticeable improvement in these symptoms. The frequency of all clinical features decreased at follow-up (Table 2).

A history of itching was reported in all AD-diagnosed children with varying levels of extent at baseline during the first visit. After six months, 342 children (80.7%) still had a history of itching. The mean and standard deviation (±SD) body surface area involvement was 34.5 (±20.6)%, ranging from 8% to 100% at baseline, and decreased to a mean (±SD) of 19.7 (±12.3)%, ranging from 2% to 64% after six months.

Sixty-one percent of children had a family atopy history, with mother 131 (30.9%), father 72 (17%), siblings 28 (6.1%), and grandparents 31 (7.3%) reporting a relationship with AD children. A history of passive smoking (i.e., where someone in the household smoked that was inhaled by the child) was recorded in 61 (14.4%) of the pediatric patients. One hundred thirty-three (31.4%) of children had a history of herbal medication use for AD treatment.

Family support was reported for 86 (20.3%) AD-affected children, and 92 (21.7%) of children had other comorbidities. The most reported comorbidity was asthma 71 (71.2%) (Table 3).

**Figure 1:**
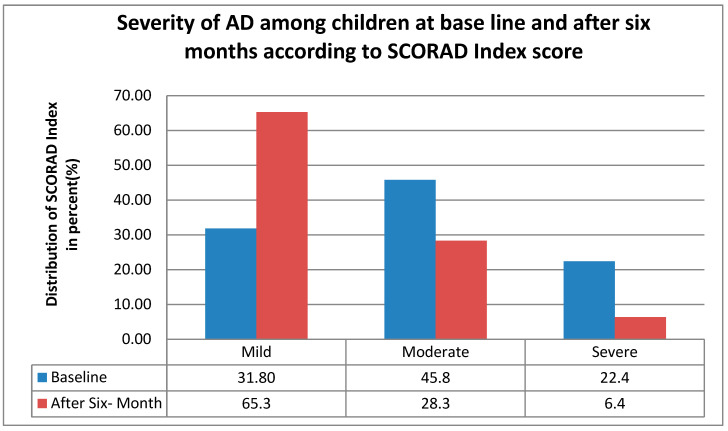
The distribution of AD severity according to the SCORAD index among children at baseline and after six months of follow-up in central and southern Ethiopia, 2024.

After a follow-up of six months, the severity of atopic dermatitis decreased, as shown in Figure 1 the mild AD increased from 31.8% (135) to 65.3% (277), while moderate and severe AD declined by 17.5% and 16%, respectively, moderate from 45.8% (194) to 28.3% (120), and severe from 22.4% (95) to 6.4% (27) indicating a clear shift toward milder disease severity over six months.

The data presented in Table 4 compare the mean scores of individual domains within the IDLQI and the CDLQI at baseline and after six months of intervention in children with AD. A significant score reduction was observed across all domains in both indices, indicating a marked improvement in QoL. For IDLQI, substantial improvements were noted in areas such as itching and scratching (from 2.05 to 1.33), mood (1.60 to 0.84), and disturbed sleep (1.27 to 0.83). These reductions suggest alleviation of symptoms and better daily functioning in infants. The total IDLQI score decreased from 13.79 ± 9.33 to 8.27 ± 7.00. Importantly, all observed changes were statistically significant with *p*-values of 0.0001.

Similarly, older children showed significant and consistent improvement in their CDLQI scores. Emotional and social domains—such as embarrassment (reduced from 1.87 to 0.93), difficulties with friendships (1.84 to 1.00), and experiences of bullying (1.16 to 0.61)—all showed marked improvement, suggesting better psychological and social well-being. Functional areas, such as sleep, participation in hobbies, and treatment-related burden, also improved over the six-month period. Overall, the total CDLQI score dropped from 13.32 ± 8.02 to 8.21 ± 7.59. All individual domain scores showed statistically significant changes (*p* < 0.0001), indicating a positive impact of the educational and treatment intervention on the children’s quality of life.

**Figure 2:**
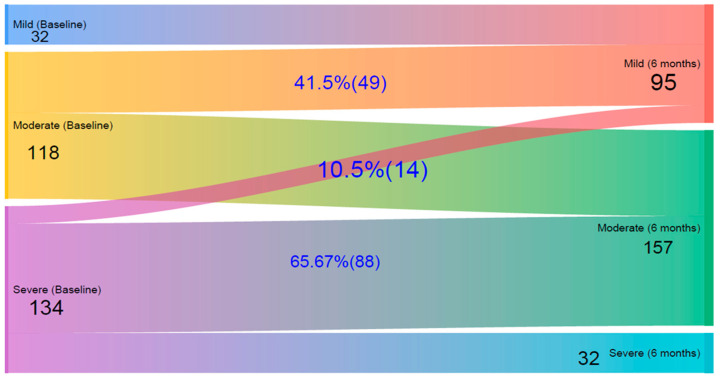
A Sankey diagram on quality of life impairment changes according to Infants’ Dermatitis Quality of Life Index (IDLQI) in children at baseline and after 6 Months follow-up at Central and Southern Ethiopia.

Figure 2 presents a Sankey diagram illustrating the changes in patients’ quality of life levels from baseline to the six-month follow-up, based on IDLQI scores. Initially, 32 children had mild, 118 had moderate, and 134 had severe QoL impairment. After six months, the number of mild cases increased to 95, reflecting improvements in many children. Among those initially classified as moderate, 41.5% improved to mild, while the rest remained moderate; importantly, none worsened to severe. Of those with severe impairment at baseline, 10.5% improved to mild, 65.7% improved to moderate, and 23.9% remained in the severe category.

**Figure 3:**
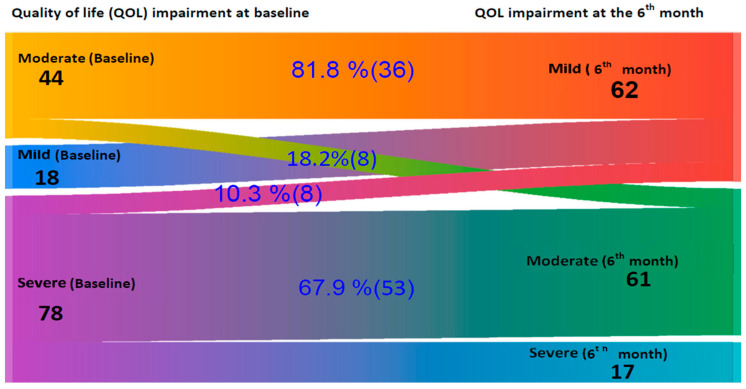
A Sankey diagram on quality of life impairment changes according to Children’s Dermatology Life Quality Index (CDLQI) in children at baseline and after 6 Months follow-up at Central and Southern Ethiopia.

Figure 3 presents a Sankey diagram that shows changes in quality of life severity (measured by CDLQI) among older children from baseline to six months. The pattern of improvement closely mirrors the findings from the IDLQI. At baseline, there were 18 mild, 44 moderate, and 78 severe impairments on QoL. After six months, the mild impairment increased to 62 due to improvements from higher severity levels. Among the moderately impaired, 81.8% (36) improved to mild, and 18.2% (8) remained moderate, with no progression to severe. Of the 78 severe cases, 10.3% (8) improved to mild, 67.9% (53) shifted to moderate, and 21.8% (17) remained in a severe category, indicating an overall shift toward milder disease severity.

### 3.3. Factors Associated with the Quality of Life of Children with Atopic Dermatitis

A univariate and multiple linear regression model assessed the relationship between sociodemographic variables, clinical characteristics, and caregiver-related variables with the Infants’ Dermatitis Quality of Life Index IDLQI) in children. Univariate linear regression analysis revealed that the child’s age, disease onset, disease duration, baseline disease severity, ∆SCORAD, child comorbidity, family support, use of herbal medication, caregiver gender, caregiver age, maternal education status, paternal education status, and occupational status all had *p*-values < 0.25 and were included in the multiple linear regression model.

The multiple linear analysis revealed that the disease severity at baseline based on the SCORAD index (95% CI: β = 0.11 [0.072–0.142]) *p* = 0.0001, change in severity after six months (β = 0.043 [0.007–0.08]) *p* = 0.019, and the number of dependent children in the family (β = −0.71 [−1.41 to −0.006]) *p* = 0.048 were identified as significantly associated variables with quality of life, as measured by the IDLQI, among children with AD (Table 5).

A univariate and multiple linear regression model was constructed to assess the relationship between sociodemographic variables, clinical characteristics, and caregiver-related variables with the children’s Dermatitis Quality of Life Index (CDLQI). The univariate linear regression analysis revealed that the disease onset, duration, baseline disease severity, ∆SCORAD, child comorbidity, caregiver age, and use of herbal medication all had *p*-values < 0.25 and were included in the multiple linear regression model.

The multiple linear analysis revealed that the onset of the disease, 95% CI: (β = 0.005 [0.001–0.01]) *p* = 0.035 and changes in severity after six months (β = 0.06 [0.001–0.116]) *p* = 0.025 were significantly associated with QoL, as measured by the IDLQI, among children with AD (Table 6).

## 4. Discussion

This study assessed the clinical characteristics, severity, and impact of atopic dermatitis (AD) on the quality of life (QoL) of children in Ethiopia. Consistent with the study’s objectives, we found that AD significantly affects both the physical and psychosocial well-being of children and imposes a measurable burden on their families. Notably, structured care and caregiver education over six months led to substantial reductions in disease severity and improvements in QoL. Key factors associated with reduced QoL include the clinical severity of the disease, the age at onset, changes in disease severity over time (SCORAD), and the number of dependent children in the household.

To our knowledge, this is one of the few prospective cohort studies from sub-Saharan Africa that quantitatively documents the burden of AD on children using validated instruments such as SCORAD, CDLQI, and IDLQI. The findings indicate the dual burden of AD not only as a chronic inflammatory skin disease but also as a condition with significant familial and emotional consequences. The study emphasizes the importance of early diagnosis, consistent follow-up, and educational interventions in alleviating disease severity and improving QoL outcomes.

The observed improvements in this study, such as reduced body surface area involvement and decreased itching, highlight the effectiveness of structured educational interventions in managing pediatric atopic dermatitis (AD) [36,37,38]. The high prevalence of a family history of atopy points to a genetic predisposition [39]. While significant exposure to passive smoking and the use of herbal remedies reflect environmental and cultural influences on the disease’s progression and treatment [40,41]. These findings emphasize the importance of early diagnosis, caregiver education, and comprehensive interventions that address both clinical and contextual factors in the care of children with AD.

In this study, we found evidence of the positive impact of routine treatment and educational interventions on the QoL in children with AD. Over six months, statistically significant improvements were observed across all domains of the IDLQI and CDLQI, underscoring the effectiveness of the intervention (both routine treatment and educational intervention) in both clinical and psychosocial measurements.

For young children, substantial improvements were noted in domains directly related to physical discomfort and functional impairment, such as itching or scratching, mood, and disturbed sleep. The reduction in mean scores for these domains reflects symptomatic relief and improved overall well-being and developmental comfort. The significant decrease in the total IDLQI score from 13.79 to 8.27 (*p* < 0.0001) further emphasizes the meaningful impact of treatment in this age group.

Likewise, older children showed significant improvements in emotional, social, and functional aspects of daily life, as reflected by lower scores in CDLQI domains such as embarrassment, difficulties with friendships, and experiences of bullying. These findings suggest that the intervention led to enhanced social integration and psychological resilience. Improvements in sleep, participation in hobbies, and a reduced treatment burden also indicate a better quality of daily life and a more manageable disease experience for patients and their families. The drop in the total CDLQI score from 13.32 to 8.21 (*p* = 0.0001) confirms the broad efficacy of the routine treatment and educational intervention. These findings align with a study in Madagascar involving young children, which reported a mean IDLQI score of 11.3 ± 3.8 for infants at baseline. After treatment, the mean IDLQI score significantly decreased to 9.3 ± 4.4. However, the mean CDLQI score among older children was 10.9 ± 3.7 for those aged 5 to 15. Following treatment, the mean CDLQI score increased to 14 ± 2.2, indicating a higher score than baseline [42]. The differences among older children may be due to cultural and environmental factors, variations in the duration of the diseases, poor adherence, the type of intervention provided, availability of family support, and severity of the diseases at baseline.

This study shows a clear shift toward milder levels of quality of life impairment among children with atopic dermatitis (AD). In infants, the number of mild cases increased from 32 to 95, with 41.5% of moderate cases and 10.5% of severe cases improving to mild. A similar pattern was seen in older children, where mild cases rose from 18 to 62, with 81.8% of moderate and 10.3% of severe cases improving to mild impairment. These changes indicate a general improvement in quality of life and a reduced disease burden. Importantly, no child progressed to a more severe category, suggesting the intervention may have helped stabilize the condition and prevent worsening.

The results of this study highlight the importance of a comprehensive management plan that includes both medical treatment and structured educational support for caregivers and patients. Such an approach addresses the clinical presentation of AD and the emotional and social dimensions that significantly affect QoL. Moreover, the consistent use of validated tools such as the IDLQI and CDLQI reinforces their value in monitoring treatment outcomes and guiding individualized care. These findings support the integration of routine, holistic care strategies in pediatric dermatology to achieve better long-term outcomes and quality of life for affected children and their families. These findings have similarities with other studies that support the idea that targeted education and consistent disease management can significantly reduce symptom burden and improve child and family well-being [36,43]. Our study aligns with previous research indicating that early treatment of moderate AD can lead to substantial quality of life improvements [1].

The age at disease onset was a significant predictor of quality of life, with children diagnosed at a younger age experiencing greater impairment as they grew older. This finding aligns with studies by Sendrasoa FA et al. (2022) and Silverberg JI et al. (2025) [42,44], emphasizing the long-term impact of early-onset atopic dermatitis. The chronic nature of the condition and its ongoing physical, emotional, and social challenges likely contribute to this cumulative burden. Additionally, baseline disease severity (SCORAD index) was strongly associated with poorer quality of life among young children, indicating that more severe AD at the start of treatment leads to greater impairment in day-to-day activities. The study’s findings are similar to those of those conducted in Serbia [1], Italy [23,26], and Turkey [45]. This finding underscores the importance of early diagnosis and aggressive treatment in children with severe AD to prevent long-term deterioration in quality of life.

In this study, nearly half of the children came from families with more than two dependents, and this was significantly associated with a poorer quality of life in younger children with atopic dermatitis, as measured by the IDLQI (*p* = 0.048). Caring for a child with AD requires substantial time and attention, which can be harder to provide in larger families with limited resources. Younger children, in particular, need more support with daily activities, increasing the caregiver’s burden. As a result, children in bigger families may receive less individualized care, negatively affecting their quality of life.

In this study, the severity of AD according to the SCORAD index score was significantly associated with quality of life impairment based on IDLQI/CDLQI among children. The change in SCORAD (∆SCORAD) over the six-month follow-up period was also significantly linked to QoL outcomes, with disease severity corresponding to improvements in the children’s overall well-being. This is consistent with the study conducted by Gazibara et al. [1], which found that lower SCORAD was associated with better quality of life initially and greater quality of life after a year of follow-up. This reinforces the value of effective disease management and educational intervention, where controlling symptoms can significantly improve children’s quality of life [46].

We also found that the baseline severity of AD, as measured by the SCORAD index, and a significant improvement in SCORAD scores after six months independently contributed to better QOL outcomes in children within our cohort. Notably, the improvement in SCORAD following the six-month follow-up was consistently associated with enhanced AD-related quality of life. These findings underscore the importance of initiating timely and individualized treatment strategies based on disease severity to optimize outcomes for children with AD [47].

Our study was conducted at comprehensive specialized and general hospitals within dermatology clinics, which may have introduced a potential limitation due to a bias toward more severe cases, as reflected by the higher proportion of moderate to severe AD cases in our cohort at baseline. Additionally, the study lacked a control group for comparison with the intervention. Furthermore, we did not analyze specific therapeutic modalities for AD or the number of medications used in its treatment. Instead, disease severity was assessed using the SCORAD index. Future studies are recommended to include control groups with large samples and detailed analyses of specific therapeutic modalities and medication use to evaluate treatment outcomes in children with AD.

## 5. Conclusions

Children with mild to severe AD showed significant improvement in AD-related quality of life. Significant improvements in quality of life were observed after six months of follow-up intervention. We noted a shift from moderate and severe impairments toward milder forms, with no worsening cases identified in our cohort. The severity of SCORAD, changes in SCORAD scores, disease onset, and the number of children in the family were independent predictors of quality of life in children with AD. Since these variables are significantly associated with quality of life, we postulate that they serve as useful markers for assessing quality of life in children with AD, thereby enabling optimal care and improved management of these cases.

## Figures and Tables

**Table 1:** The Sociodemographic Characteristics of Children Diagnosed with Atopic Dermatitis and Their Families in Central and Southern Regional States of Ethiopia, 2024.

Variables	Category	Frequency	Percent
Sex	Female	193	45.5
	Male	231	54.5
Age of Children	≤4 yrs	284	67
	5–10 yrs	95	22.4
	11–16 yrs	455	10.6
Residence	Rural	171	40.3
	Urban	253	59.7
Family Occupation	Employee	167	39.4
	Farmer	54	12.7
	Housewife	70	16.5
	Merchant	133	31.4
Monthly Income in ETB	≤5000 ETB	212	50
	5001–10,000 ETB	157	37
	≥10,001 ETB	55	13
Education status Mother	No formal education	97	22.9
	Primary school	92	21.7
	Secondary school	66	15.6
	Tertiary (Diploma and above)	169	39.9
Education status Father	No formal education	73	17.2
	Primary school	78	18.4
	Secondary school	73	17.2
	Tertiary (Diploma and above)	200	47.2
Marital status caregiver	Widowed/divorced	22	5.2
	Married	373	88.0
	Single	29	6.8
Age of caregiver	18–24 yrs	38	9.0
	25–34 yrs	266	62.7
	≥35 yrs	120	28.3
Caregiver gender	Female	292	68.9
	Male	132	31.1
Number of children	≤2	216	50.9
	3–4	135	31.8
	≥5	73	17.2

ETB = Ethiopian Birr, Yrs = Years.

**Table 2:** Clinical Features of AD-Affected Children at Baseline and Six-Month Follow-Up in Central and Southern Regional States of Ethiopia, 2024.

Clinical Feature	Baseline (n, %)	6-Month Follow-Up (n, %)
Dry skin (xerosis)	383 (90.3%)	308 (72.6%)
Redness (erythema)	363 (85.6%)	254 (59.9%)
Scratch marks (excoriation)	340 (80.2%)	274 (64.6%)
Lichenification	309 (72.9%)	237 (55.9%)
Swelling (edema)	267 (63.0%)	194 (45.8%)
Oozing/crusting	231 (54.5%)	174 (41.0%)

**Table 3:** Clinical Characteristics Profile Among Atopic Dermatitis Diagnosed Children and Their Families in Central and Southern Ethiopia, 2024.

Variables	Category	Frequency (N)	Percent (%)
Age of onset	<2 years	306	72.2
	≥2 years	118	27.8
How long since diagnosed	<1 year	287	67.7
	≥1 year	137	32.3
Blood relation with caregiver	Mother	265	62.5
	Father	114	26.9
	Siblings	24	5.7
	Other	21	5.0
A smoking person in the family	No	363	85.6
	Yes	61	14.4
Herbal Medication Use	No	291	68.6
	Yes	133	31.4
Family Atopy history	No	164	38.7
	Yes	260	61.3
Family support	No	338	79.7
	Yes	86	20.3
Comorbidity of child	No	332	78.3
	Yes	92	21.7
Comorbidity of caregiver	No	306	72.2
	Yes	118	27.8
Comorbid diseases of child	Asthma	71	71.7
	Diabetes mellitus	19	19.2
	Other	9	9.1

**Table 4:** Mean values (standard deviation) Scores for Individual Domains of the Infant Dermatitis Quality of Life Index (IDLQI) and Children’s Dermatology Life Quality Index (CDLQI) at Baseline and after 6 Months follow-up at Central and Southern Ethiopia.

Mean (±SD) Scores for Individual Domains of the Infant Dermatitis Quality of Life Index (IDLQI) and Children’s Dermatology Life Quality Index (CDLQI) at Baseline and After 6 Months							
Items	Baseline	After 6 Months	*p* Value		Baseline	After 6 Months	*p* Value
IDLQI	Mean (±SD)	Mean (±SD)		CDLQI	Mean (±SD)	Mean (±SD)	
1. Itching or scratching	2.05 (0.73)	1.33 (0.69)	<0.0001	1. Itching or scratching	1.71 (0.79)	1.14 (0.73)	<0.0001
2. Mood	1.60 (0.95)	0.84 (0.7)	<0.0001	2. Embarrassment	1.87 (0.82)	0.93 (0.72)	<0.0001
3. Time to get to sleep	1.46 (1.0)	0.89 (0.78)	<0.0001	3. Affected friendships	1.84 (0.89)	1.00 (0.8)	<0.0001
4. Sleep disturbance	1.27 (1.02)	0.83 (0.73)	<0.0001	4. Clothing	1.59 (0.84)	0.91 (0.78)	<0.0001
5. Impaired playing	1.19 (0.97)	0.67 (0.62)	<0.0001	5. Playing/hobbies	1.32 (0.98)	0.74 (0.82)	<0.0001
6. Interference with family activities	1.36 (0.93)	0.79 (0.70)	<0.0001	6. Swimming/sports	1.26 (0.78)	0.73 (0.75)	<0.0001
7. Problem at mealtimes	1.23 (0.90)	0.78 (0.73)	<0.0001	7. School/holiday	0.95 (0.83)	0.59 (0.73)	<0.0001
8. Problems caused by the treatment	1.09 (0.99)	0.68 (0.72)	<0.0001	8. Teasing/bullying	1.16 (0.93)	0.61 (0.68)	<0.0001
9. Uncomfortable dressing	1.27 (0.94)	0.77 (0.70)	<0.0001	9. Affected sleep	1.19 (0.89)	0.76 (0.77)	<0.0001
10. Problem at bath time	1.27 (0.89)	0.70 (0.63)	<0.0001	10. Problem with treatment	1.38 (1.10)	0.81 (0.81)	<0.0001
Total IDLQI score	13.79 (9.33)	8.27 (7)		Total CDLQI score	13.32 (8.02)	8.21 (7.59)	

**Table 5:** Linear Regression Analysis of Factors Associated with Children’s Quality of Life as Measured by the Infants’ Dermatitis Quality of Life Index After Six Months of Follow-Up at Central and Southern Ethiopia.

Independent Variables	Univariate Linear Regression		Multiple Linear Regression	
	Beta Coefficient (95% CI)	*p* Value	Beta Coefficient (95% CI)	*p* Value
Child’s age (years)	0.34 (−0.135–0.82)	0.159		
Child’s gender (Male versus Female)	0.83 (−0.53–1.79)	0.127		
Residence (Urban vs. rural)	−0.397 (0.47–1.485)	0.69		
Onset of the disease	0.016 (0.003–0.03)	0.014		
Duration of AD (years)	0.023 (0.011–0.034)	0.0001		
Family Atopy history (Yes versus no)	0.16 (−1.25–0.92)	0.77		
SCORAD at baseline	0.135 (0.11–0.16)	0.0001	**0.11 (0.072–0142)**	**0.0001** *
∆SCORAD	0.127 (0.098–0.155)	0.0001	**0.043 (0.007–0.08)**	**0.019**
Child comorbidity (Yes versus no)	2.5 (1.27–3.74)	0.0001		
Family support (Yes versus no)	1.03 (−0.33–2.40)	0.138		
Herbal medication use (Yes versus no)	0.85 (−0.28–1.97)	0.14		
Smoking exposure (Yes versus no)	1.10 (−0.097–2.29)	0.072		
Caregiver gender (Male versus Female)	−1.46 (−2.6–−0.31)	0.013	0.08 (−0.02–0.17)	
Caregiver Age (years)	0.08 (−0.02–0.17)	0.12		
Maternal level of education	0.77 (0.33–1.1.2)	0.001		
Parental level of education	0.70 (0.24–1.15)	0.003		
Number of children in the Family	0.45 (−0.31–1.21)	0.24	**−0.71 (−1.41–−0.006)**	**0.048** *
Family occupation	0.26 (−0.14–0.66)	0.20		
Family monthly income	−1.13 (−1.9–−0.38)	0.003		
Marital status	0.09 (−1.73–1.90)	0.92		

∆SCORAD: SCORAD at baseline − SCORAD after six months of follow-up, CI: confidence interval, AD: atopic dermatitis, SCORAD: scoring AD index, * Symbol indicates *p* value < 0.05.

**Table 6:** Linear Regression Analysis of Factors Associated with Children’s Quality of Life as Measured by the Children’s Dermatology Life Quality Index After Six Months of Follow-Up at Central and Southern Ethiopia.

Independent Variables	Univariate Linear Regression	*p* Value	Multiple Linear Regression	*p* Value
	Beta Coefficient (95% CI)		Beta Coefficient (95% CI)	
Child’s age (years)	0.09 (−0.09–0.28)	0.31		
Child’s gender (Male versus Female)	−0.33 (−1.63–0.97)	0.62		
Residence (Urban vs. rural)	0.16 (−1.13–1.46)	0.80		
Onset of the disease	0.009 (0.004–0.013)	0.0001	**0.005 (0.001–0.01)**	**0.035** *
Duration of AD (years)	0.006 (0.001–0.010)	0.029		
Family Atopy history (Yes versus no)	0.73 (−0.58–2.04)	0.273		
SCORAD at baseline	0.06 (0.027–0.091)	0.000		
∆SCORAD	0.086 (0.046–0.127)	0.000	**0.06 (0.001–0.116)**	**0.027** *
Child comorbidity (Yes versus no)	1.62 (0.043–3.20)	0.044		
Family support (Yes versus no)	0.62 (−0.87–2.11)	0.413		
Herbal medication use (Yes versus no)	1.73 (0.35–3.11)	0.015		
Smoking exposure (Yes versus no)	0.23 (−1.28–1.73)	0.77		
Caregiver gender (Male versus Female)	−0.72 (−2.07–0.64)	0.30		
Caregiver Age (years)	0.06 (−0.04–0.16)	0.226		
Maternal level of education	0.11 (−0.43–0.64)	0.69		
Parental level of education	0.054 (−0.48–0.59)	0.843		
Number of children in the Family	−0.135 (−0.49–0.223)	0.46		
Family occupation	0.177 (−0.365–0.72)	0.52		
Family monthly income	0.042 (−0.85–0.93)	0.93		
Marital status	0.22 (−1.29–1.73)	0.77		

AD: atopic dermatitis, ∆SCORAD: (SCORAD at baseline − SCORAD after six months of follow-up), CI: confidence interval, SCORAD: scoring AD index * Symbol indicates *p* value < 0.05.

## Data Availability

The raw data supporting the conclusions of this article will be made available by the corresponding author without undue reservation.
